# Present-Day Genetic Structure of Atlantic Salmon (*Salmo salar*) in Icelandic Rivers and Ice-Cap Retreat Models

**DOI:** 10.1371/journal.pone.0086809

**Published:** 2014-02-03

**Authors:** Kristinn Olafsson, Christophe Pampoulie, Sigridur Hjorleifsdottir, Sigurdur Gudjonsson, Gudmundur O. Hreggvidsson

**Affiliations:** 1 Faculty of Life and Environmental Sciences, University of Iceland, Reykjavik, Iceland; 2 Marine Research Institute, Reykjavík, Iceland; 3 Institute of Freshwater Fisheries, Reykjavík, Iceland; 4 Genetics, Matis Ltd., Reykjavík, Iceland; Swedish Museum of Natural History, Sweden

## Abstract

Due to an improved understanding of past climatological conditions, it has now become possible to study the potential concordance between former climatological models and present-day genetic structure. Genetic variability was assessed in 26 samples from different rivers of Atlantic salmon in Iceland (total of 2,352 individuals), using 15 microsatellite loci. *F*-statistics revealed significant differences between the majority of the populations that were sampled. Bayesian cluster analyses using both prior information and no prior information on sampling location revealed the presence of two distinguishable genetic pools - namely, the Northern (Group 1) and Southern (Group 2) regions of Iceland. Furthermore, the random permutation of different allele sizes among allelic states revealed a significant mutational component to the genetic differentiation at four microsatellite loci (SsaD144, Ssa171, SSsp2201 and SsaF3), and supported the proposition of a historical origin behind the observed variation. The estimated time of divergence, using two different ABC methods, suggested that the observed genetic pattern originated from between the Last Glacial Maximum to the Younger Dryas, which serves as additional evidence of the relative immaturity of Icelandic fish populations, on account of the re-colonisation of this young environment following the Last Glacial Maximum. Additional analyses suggested the presence of several genetic entities which were likely to originate from the original groups detected.

## Introduction

For decades, several types of genetic markers have been used to define populations' boundaries in a multitude of species. An alternative approach has only been recently developed due to better understanding of past climatological conditions (i.e. the potential concordance between former climatological models and present-day genetic structure). Indeed, contemporary genetic patterns are known to be the result of both present-day and historical factors [1]. One of the most fundamental premises is that glaciation and inter-glaciation periods of the Pleistocene have shaped the genetic structure of many contemporary species. Present-day genetic structure of populations of a species inhabiting past refugium show a higher level of genetic diversity than those inhabiting formerly glaciated regions, due to range expansion and genetic drift following deglaciation [2], [3]. In the last few decades, examples of concordance between climatological history and present-day genetic structure have been reliably noted within taxa inhabiting freshwater or land habitats [4]–[12], depending on the availability of climatological information and models. Accurate and precise models of environmental conditions and ice-sheet covers have only recently become available for the North Atlantic [13]–[17], and as a consequence the effect of glaciation on the genetic structure of marine species has only recently been investigated - especially so in the North Atlantic Ocean [13], [18]–[24]. This is also true for the climatological history around Iceland, which has been only recently resolved [17], [25]–[27].

Although phylogeography studies related to anadromous fish such as the Atlantic salmon [28], [29] sometimes consider the effect of the Pleistocene on contemporary genetic patterns [30]–[32], so far no correlation has been made between the divergence time and climatological history of this species. The Atlantic salmon is a philopatric species exhibiting a complex biological cycle that includes spawning in rivers, a freshwater juvenile phase followed by subsequent oceanic feeding migrations, and a high fidelity to natal rivers [33]. This complex life-cycle is often considered the source of reproductive isolation, which facilitates the evolution and persistence of locally adapted populations [34]. In the last 20 years, genetic studies regarding the Atlantic salmon have flourished, in that they have confirmed that populations are highly structured both between and within rivers systems [35]–[38], and that the associated genetic pattern was usually temporally stable [39]–[41]. In Icelandic rivers, the only genetic study performed using allozyme data suggested restricted gene flow among rivers as well as within large river systems [35].

Although the complex biological cycle of the Atlantic salmon has been determined to be the primary source of potential genetic signal, another major factor that could have been the origin of the present-day genetic pattern in the Atlantic salmon is the geological and climatological history of the North Atlantic Ocean. Past geological and climatological history has, indeed, been shown to be at the origin of genetic structure and patterns of several land or marine organisms [3], [10], [42]–[44]. Deglaciation following the Last glacial maximum (LGM) may, therefore, illustrate the process of river colonization, which would in turn partly explain the genetic structure of later generations of Atlantic salmon in Iceland. LGM is usually reported between 21–17 cal. kyr BP (calibrated years before the present) [17], [25]–[27].

The objective of the present study was, therefore, to investigate the genetic structure of Atlantic salmon collected from 26 Icelandic rivers using 15 neutral markers, and to assess whether the genetic pattern observed could be attributed to the climatological history of Iceland.

## Materials and Methods

### Sampling and genotyping

**Figure 1:**
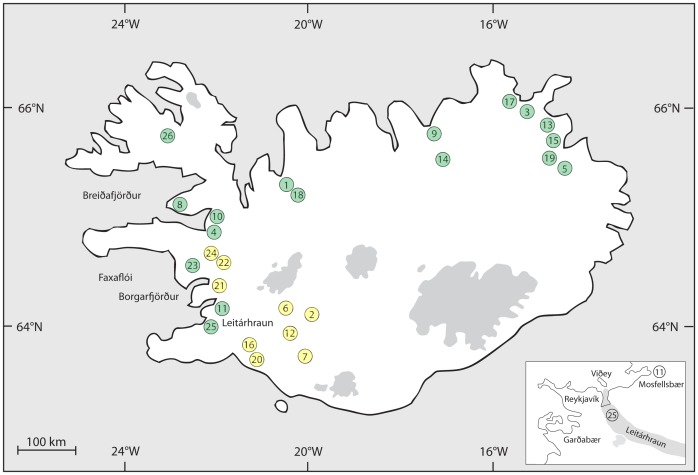
Geographical distribution of 26 sampling locations of Atlantic salmon in Icelandic rivers. Numbers refer to the samples codes in Table 2 with those in green belonging to Group 1 and those in yellow belonging to Group 2. The insert shows the pathway of the lava field Leitárhraun forming the present river channel of the river Ellidaa (25).

A total of 2,352 salmon parr were sampled by electro fishing from 400–500 m stretches at 26 rivers in 2002 (Ellidaa, (25)) and 2004 (all others) (Figure 1, Table 1). Each sample consisted of 54–94 parr, comprising 3 or 4 year classes ranging from 1 to 5 years old. All samples were collected by the Institute of Freshwater Fisheries with full authority from the Directorate of Fisheries in accordance with Icelandic law on salmon and trout fishing number 61/2006, article 26, subparagraph two [45]. All fish were put to death in accordance with Icelandic law on “*Animal Welfare number 15/1994, article 14*” [46]. Samples were taken from across the species range in Iceland – the lack of samples obtained from the southern and eastern coast is due to the fact that the rivers in this region are mostly turbid, glacial-fed rivers, which are unsuitable salmon habitats [47]. Genotype information was retrieved from 15 microsatellite loci (Table S1), and both PCR condition and genotyping procedures were performed as described in Olafsson *et al.* [48].

**Table 1:** Sampling code, sampling location (Latitude: Lat; Longitude: Long), river names (river), groups used in STRUCTURE, number of full siblings removed from sample and number of sibling groups, and genetic diversity details (n is the number of individuals assessed, *Ar* is allelic richness, *H_E_* is unbiased gene diversity, *F_IS_* the inbreeding coefficient within subpopulations, HWE is the probability of Hardy-Weinberg equilibrium).

#	River	Abreviation	Lat	Long	River system	Structure Goups	Year	Full Sibs/groups	n	*Ar*	*H_E_*	*F_IS_*	HWE
1	Blanda	Bla	65°31,120′N	−19°52,752′W	Blanda	G1	2004	13/8	80	8.551	0.720	−0.0320	0.1403
2	Dalsa	Dal	64°16,185′N	−20°11,174′W	Olfus	G2	2004	9/4	81	7.741	0.681	−0.0290	0.0740
3	Hafralonsa	Haf	66°6,096′N	−15°25,535′W	Hafralonsa	G1	2004	10/5	78	8.987	0.725	−0.0030	0.0723
4	Haukadalsa	Hauk	65°3,004′N	−21°41,257′W	Haukadalsa	G1	2004	1/1	87	9.027	0.720	−0.0130	0.0694
5	Hofsa	Hof	65°37,135′N	−15°3,826′W	Hofsa	G1	2004	2/2	91	8.612	0.710	−0.0020	0.7014
6	Hvita Efst	Hvit	64°16,516′N	−20°11,722′W	Olfus	G2	2004	11/5	76	8.317	0.675	−0.0220	0.0070
7	Kalfa	Kal	64°2,223′N	−20°18,119′W	Þjorsa	G2	2004	8/7	81	6.782	0.630	−0.0320	0.2981
8	Krossa	Kro	65°15,617′N	−22°17,916′W	Krossa	G1	2004	24/7	68	8.206	0.688	−0.0290	0.1240
9	Laxa Adaldal	Ladl	65°57,594′N	−17°24,224′W	Laxa Adaldal	G1	2004	13/5	72	7.613	0.681	−0.0270	0.0158
10	Laxa Dolum	Ldol	65°8,139′N	−21°36,474′W	Laxa Dolum	G1	2004	0/0	94	8.916	0.697	0.0240	0.0158
11	Leirvogsa	Leir	64°11,551′N	−21°40,050′W	Leirvogsa	G1	2004	11/6	82	8.345	0.690	0.0030	0.9092
12	Litla Laxa	Llax	64°8,054′N	−20°19,673′W	Olfus	G2	2004	40/1	54	8.148	0.654	−0.0100	0.5292
13	Midfjardara	Mid	66°2,215′N	−15°6,731′W	Midfjardara	G1	2004	12/3	71	8.401	0.693	−0.0270	0.1370
14	Reykjadalsa	Rey	65°44,798′N	−17°23,251′W	Laxa Adaldal	G1	2004	16/5	74	8.027	0.673	0.0000	0.1413
15	Sela	Sel	65°49,471′N	−14°50,824′W	Sela	G1	2004	16/2	74	9.232	0.734	−0.0400	0.3628
16	Sog	Sog	64°3,698′N	−20°59,353′W	Olfus	G2	2004	2/2	88	8.847	0.710	−0.0030	0.4081
17	Svalbardsa	Sval	66°11,182′N	−15°43,459′W	Svalbardsa	G1	2004	21/3	66	8.703	0.679	−0.0130	0.0915
18	Svarta	Svar	65°31,307′N	−19°49,468′W	Blanda	G1	2004	13/8	71	8.408	0.704	0.0190	0.0151
19	Vesturdalsa	Vest	65°42,043′N	−15°0,037′W	Vesturdalsa	G1	2004	30/6	62	7.703	0.695	−0.0570	0.5365
20	Olfusa	Olf	63°57,124′N	−20°58,991′W	Olfus	G2	2004	8/4	84	8.920	0.695	−0.0300	0.6443
21	Grimsa	Grim	64°32,160′N	−21°19,079′W	Hvita	G2	2004	7/5	80	8.128	0.718	−0.0240	0.0158
22	Kjarra	Kjar	64°45,271′N	−21°7,115′W	Hvita	G2	2004	5/3	87	8.025	0.693	−0.0110	0.1363
23	Langa	Lag	64°39,835′N	−21°53,087′W	Langa	G1	2004	1/1	91	8.618	0.696	0.0130	0.1632
24	Litla Thvera	Lthe	64°47,057′N	−21°19,481′W	Hvita	G2	2004	11/4	69	7.850	0.708	−0.0090	0.2650
25	Ellidaa	Ell	64°7,339′N	−21°50,413′W	Ellidaa	G1	2002	10/7	82	7.811	0.675	0.0100	0.2372
26	Laugadalsa	Lau	65°58,129′N	−22°39,802′W	Laugadalsa	G1	2004	5/5	85	8.586	0.707	0.0050	0.4079

### Statistical analyses

Samples from early life stages contain only the genetic material of successful breeders -often of a single year- and may be biased towards particular families [49]. Full-sibling and half-sibling groups were identified within each sample using COLONY 1.2 [50]. After identifying any full sibling groups nestled within half-sibling families, we used a trial and error based method with HWE as a benchmark with which to assess the appropriate stringency level required to eliminate the sampling of siblings' effect [49]. After repeated attempts of various combinations of tolerance level, we found that, by allowing only two full siblings and six half-siblings in each family, we were able to reduce the sib-ship effects sufficiently with reference to HWE. After the reduction of full-and half sibling groups in our samples, genetic variability was assessed as number of alleles, and the observed (*H*_O_) and expected (*H*_E_) heterozygosities and *F*-statistics [51] were calculated in GENETIX 4.05.2 [52] for each sample. The samples were also tested for conformation to Hardy-Weinberg expectations (HWE) with GENEPOP [53]. Genetic diversity was further quantified using FSTAT 2.9.3 [54] to calculate Nei's unbiased diversity (*H*_S_, average expected heterozygosity) of individual samples. The significance of pairwise *F_ST_* (for all pairs of populations) was calculated in ARLEQUIN 3.5.1.3 [55] using 10,000 permutations, and a principal coordinates analysis (PCA) was performed in GENALEX 6.1 [56] based on *F_ST_* values. To detect loci under directional selection we used the program LOSITAN [57] to generate 100,000 simulated loci, providing an expected neutral distribution of *F_ST_* values and an estimate of p-value for each locus.

In order to infer genetic ancestry and to identify subgroups that have different genotype patterns, multi-locus genotypes were analysed by a model-based clustering algorithm with STRUCTURE 2.3 [58]. Three runs were performed for each K value, ranging from 1 to 26. We chose 500,000 iterations of the Gibbs sampler after a burn-in of 50,000 iterations, applying a model that allows for admixture whilst implementing a correlated allele frequency. To determine the most likely hierarchical level of genetic structure, we plotted values of LnP(D) (the log probability of data) for each K and estimated ΔK statistics, which is based on the rate of change in LnP(D) between successive K values [59]. We also analysed the data using the admixture model with the LOCPRIOR setting, which considers location information. STRUCTURE was also used to calculate the *F* value for the implemented *F*-model for correlated allele frequencies which can be used in a historical inference [60]. The model for correlated allele frequencies developed by Falush *et. al* [60] assumes that, “*K populations represented in our sample have each undergone independent drift away from ancestral allele frequencies at rates parameterized by F1, F2, F3…Fk respectively*”. These F1 to Fk values can be used to make evolutionary inferences (see [60], page 1570).

Evanno *et al.* [59] suggests that STRUCTURE tends to only capture the major structure in the data, although a subsequent STRUCTURE analysis performed on each identified cluster can potentially demonstrate a more intricate population structure within these clusters. This analysis was performed using a modified “hierarchical STRUCTURE analysis” as outlined by Vähä *et al.* [61] and Warnock *et al.* [62]. Calculations were made applying the same parameters for the Gibbs sampler and the burn-in iteration, but with K ranging from one to ten; ΔK statistics were consequently calculated to decide the hierarchical structure at each level [59]. We therefore conducted a hierarchical analysis by performing similar Structure runs on each detected group (K) containing several samples. The estimated number of group (K) was based on ΔK statistics and changes in the pattern of LnP(D) values.

A hierarchical analysis of molecular variance (AMOVA) was performed using Arlequin [55] to quantify the degree of differentiation between the post-hoc defined regions according to the Bayesian approach in STRUCTURE, i.e. for K = 2. A consensus neighbour joining tree of pairwise D_A_ [63] between all samples was calculated and constructed using the software POPULATIONS 1.2.28 [64]. Genetic diversity indices, such as *H*s and Allelic richness, were then compared between groups of the uppermost hierarchical structure based on ΔK from STRUCTURE [58] using FSTAT [54].

To assess the potential impact of mutation *vs.* drift on the genetic pattern detected, we performed the allele-size randomization test [65] implemented in SPAGeDi 1.3 [66]. The objective of this analysis was to test whether *F*_ST_ = *R*_ST_, where *R*_ST_ is a stepwise mutation model (SMM)-based measure of genetic differentiation informed by microsatellite allele size variance. This is analogous to *F*_ST_, and unbiased with respect to any differences in sample size between populations or any differences in variance between loci. When mutations contribute little to genetic differentiation, estimates of differentiation using *F*_ST_ and *R*_ST_ should show similar results. On the other hand, if stepwise-like mutations have contributed significantly to divergence, *R*_ST_ should demonstrate a larger differentiation than that of *F*_ST_. Potential historical signatures in the genetic data were therefore assessed by permutating allele sizes at each microsatellite locus among allelic states (max 20,000 replicates) to simulate distribution of R_ST_ values (ρR_ST_) with 95% confidence intervals (CI).

To further clarify evolutionary history, the time of divergence was approximated using two different ABC approaches. First, the time of divergence was approximated using the Approximate Bayesian Computation (ABC) implemented in DIYABC v1.0.4.46 [67]. ABC is a Bayesian-based approach where the posterior distributions of the parameters in the model are predicted by replacing the calculations of the likelihood (probability of observed data given the values of the model parameters) by a measure of similarity between observed and simulated data [67]. The ABC method can be described as three sequential steps - the first step (*simulation*) simulates many multilocus data sets with characteristics similar to the observed data set; the second step (*rejection*) compares the simulated data set to the observed data set and retains simulations that are arbitrarily close to the observations whilst rejecting others; the final step (*estimation*) then estimates posterior distributions of parameters through locally weighted linear regression on the summary statistics associated with the retained simulations [68]. The tested scenario was that two populations of size N1 and N2 have diverged *t* generations in the past from an ancestral population of size N1+N2, assuming a stepwise mutation model with no indel mutations and a mutation rate μ = 10^−4^. In the first step, 1,000,000 datasets were simulated, and 1% of the simulated data sets that most closely fit the observed data were used to inform the posterior distribution of parameter values. The uniform prior distributions used in the DIYABC analyses ranged from 0–20,000 for both N1 and N2 and 0–50,000 for *t*. Secondly, the time of divergence was approximated using the popABC software [69] where we evaluate a model of an ancestral population of size N_A_ that splits at time T in the past to give two populations of size N_1_ and N_2_. Since the time of splitting, immigration occurs at rates m_1_ and m_2_, where immigrants into one population are drawn from the other population. The mean of mean mutation rate among loci was assumed to 5×10^−4^, normally distributed with a standard deviation of 5×10^−4^. A mean mutation rate of 5×10^−4^ is commonly assumed in demographic models [70], but is a little lower than pedigree-based estimates for autosomal microsatellites in humans [71], [72]. Five million data points from the joint distribution of parameters and summary statistics were simulated, and the 50,000 points closest to each target set of summary statistics were used for regression-adjustment [73].

## Results

### Genetic variability and siblings

A total of 324 individuals (i.e. 13.78% of the total database) were discarded due to sibling effects within the sampling, ranging from 40 in Litla Laxa (12) to none in Laxa Dolum (10) (table 1). A total of 205 alleles were observed across the 15 loci, ranging from two alleles in Ssa14 and SsaD486 to 32 in SsaD144. The locus SsaD486 is usually considered a continental analysis marker, and one that is therefore poorly suited to small-scale genetic analysis, and not polymorphic within eastern populations of Atlantic salmon [74]. However, it has been suggested to be particularly useful in discriminating between brown trout (*Salmo trutta*), salmon, and hybrids thereof [75]. Hybridization between the two taxa has been widely documented [76]–[78]. Based on this locus, two individuals were discarded from our samples as hybrids of salmon and trout, showing the exact allele sizes for SsaD486 as described in Perrier *et al.* [75]. This locus was monomorphic in 18 of the 26 populations, and the frequency of the second allele was 0.0029 overall, and thus we chose to discard it from subsequent analyses.

Only one sample deviated from HWE (i.e. the river Hvita Efst (6) (Table 1)); this was mainly due to locus Ssa2216 being out of HWE in that sample. The overall genetic variability of samples, as measured by gene diversity (*H*_S_) and allelic richness (*A*_R_), ranged from 0.630–0.730 and 6.78–9.23, respectively (Table 1).

### Genetic differentiation

An overall *F*_ST_ value of 0.057 was observed on a sample level. The degree of genetic differentiation between sampling locations, as estimated by *F*_ST_, ranged between 0.006 and 0.140 (Table S2). Highly significant genetic differentiations (Fisher's exact test P<0.01) were observed between all samples except: Olfusa (20), Sog (16), Blanda (1), and Svarta (18). A PCA based on the *F_ST_* is presented in Figure S1. No signs of directional or balancing selection were identified at the 14 loci among the two populations (Figure S2).

**Figure 2:**
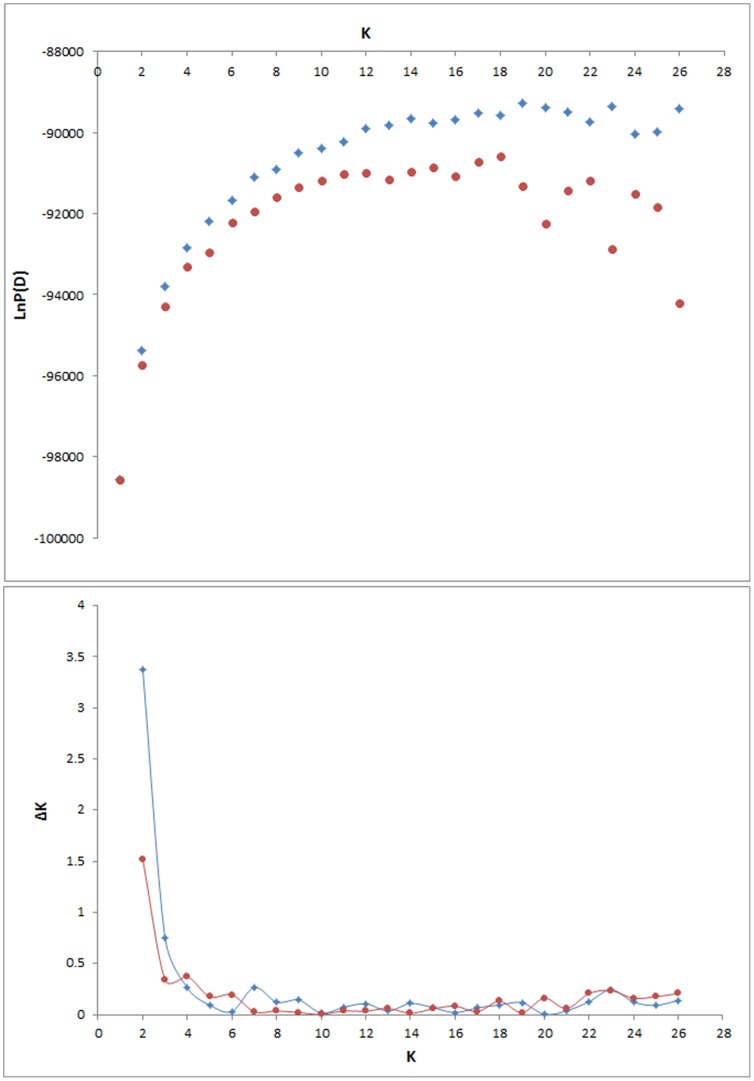
Uppermost hierarchical structure based on ΔK. (A) Estimated likelihood, LnP(D) for values of K ranging from 1 to 26. The mean LnP(D) for each K over 3 runs are represented by red solid dots (•) and a blue diamond when considering location information (◊). (B) ΔK calculated according to Evanno *et al.* [59]. The modal value of this distribution corresponds to the true K(*) or the uppermost level of structure, here two clusters.

**Figure 3:**
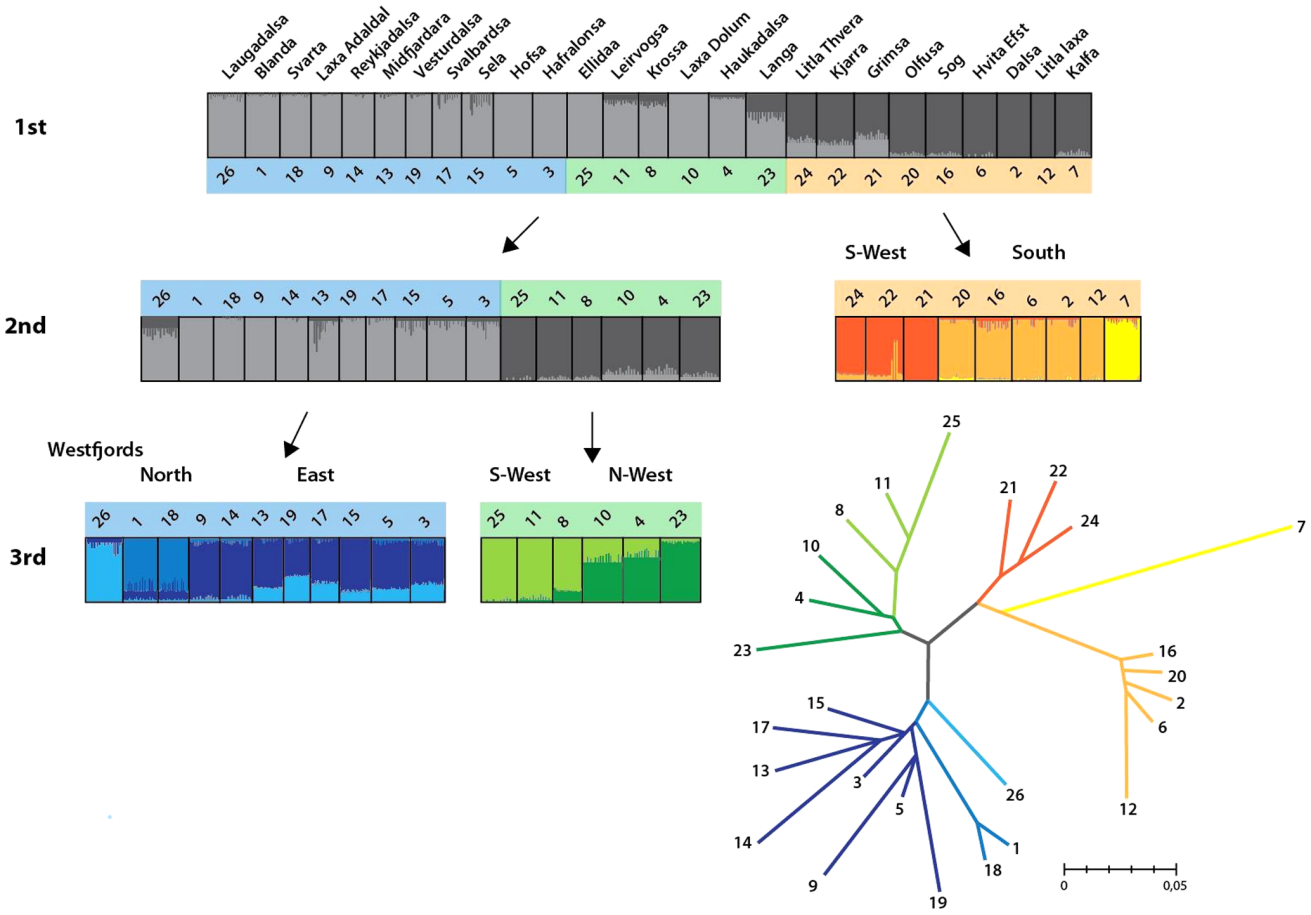
Population structure as estimated from 3 levels of hierarchical STRUCTURE analysis (Pritchard *et al.* [58]). Populations are represented by vertical lines which are partitioned into shaded segments representing the populations estimated membership of the two or three possible clusters defined in the STRUCTURE run. Rows represent the hierarchical approach with subsets of populations separated and re-analysed. The subsets used are delineated below each plot. A consensus neighbour joining tree of pairwise D_A_ [63] between all samples is also presented; colors of the branches/groups correspond to the different groups detected in the hierarchical structure analysis.

The Bayesian cluster analysis and subsequent calculations of ΔK showed that the most likely number of genetically distinguishable groups (*K*) was two, both with (Ln *P*(*D*)±S.D = −95402±462) and without location information (Ln *P*(*D*)±S.D = −95725±922) (Figure 2). The first group (Group 1) was composed of samples from the northwest, north, and east rivers: 1; 3–5; 8–11; 13–15; 17–19; 23; 25–26 (Figure 1 green). The second (Group 2) was composed of samples from the southwest and south of Iceland: 2; 6–7; 12; 16; 20–22; 24 (Table 1, Figure 1 yellow). The average F-values as outputted by STRUCTURE for the K = 2 runs were 0.032 and 0.060 for the two groups, Group 1 and Group 2 respectively. The population structure revealed by the hierarchical clustering method is outlined in Figure 3. Initial partitioning of the dataset at the 1^st^ level using *K* = 2 separated the data into the two previously described groups, Group 1 and Group 2. Further levels of analysis within these and subsequent hierarchical groupings resulted in geographically comprehensive groupings at each level, leading to a total of eight major regional groupings at the 2^nd^ and 3^rd^ level of analysis (Figure 3). Branches on the NJ-plot tree of the genetic distance D_A_ [63] corresponded to the colours of the eight groups identified with hierarchical structure analysis (Figure 3). The clustering assignment results for *K* = (2, 3, and 4), with and without location information, are presented in Figure S3. The AMOVA between groups detected by the Bayesian clustering method corresponded to the historical origin in the data. The observed level of genetic differentiation (*F*_ST_) was 0.072 between the two groups (Table S3).

In addition, when the genetic diversity of Group 1 and Group 2 were compared, no significant trend was observed, although the gene diversity (Hs) and allelic richness (Ar) tended to be slightly higher in Group 1 than in Group 2, (Ar = 8.456 and 8.084, Hs = 0.699 and 0.685, respectively).

### Historical origin

The random permutation of different allele sizes among allelic states at each locus revealed that estimates of *R*_ST_ were significantly larger than the 95% CI range of the ρ*R*_ST_ values at four microsatellite loci (SsaD144, Ssa171, SSsp2201 and SsaF3), suggesting a mutational component to genetic differentiation. *R*_ST_ was also larger than the ρ*R*_ST_ values at SSsp2210, but not significantly. When considering the two groups detected with the Bayesian method, the overall estimate of *R*_ST_ was also significantly larger than the 95% CI range of the ρ*R*_ST_ values (Table 2).

**Table 2:** Mean single locus and multilocus estimates of *R*_ST_, θ_ST_ and ρ*R*_ST_ (95% distribution of central values in parentheses) between 26 samples of Atlantic salmon following 20 000 allele permutations (Hardy *et al.* [65]).

	θ_ST_	*R*_ST_	ρ*R*_ST_ (95% range)
Ssa14	0.054	0.054	0.0541 (0.0541–0.0541)
Ssa171*	0.073	0.138	0.0716 (0.0231–0.1446)
SSa197	0.052	0.046	0.0526 (0.0178–0.1231)
Ssa202	0.045	0.042	0.0453 (0.0191–0.0752)
Ssa289	0.075	0.047	0.0673 (0.0319–0.0855)
Sp1605	0.066	0.023	0.0636 (0.0298–0.1007)
Sp2201*	0.036	0.065	0.0360 (0.0137–0.0675)
SSsp2210	0.081	0.094	0.0822 (0.0304–0.1546)
Sp2216	0.057	0.020	0.0554 (0.0253–0.0881)
SSsp3016	0.046	0.038	0.0473 (0.0201–0.0885)
SsaD144**	0.053	0.132	0.0521 (0.0148–0.1044)
Ssa157	0.032	0.002	0.0311 (0.0125–0.0597)
SsaF43*	0.079	0.109	0.0720 (0.0231–0.1114)
SSspG7	0.085	0.075	0.0829 (0.0271–0.1550)
Multilocus	0.058	0.035	0.0312 (0.0128–0.0596)
Group 1 vs Group2*	0.034	0.091	0.0356 (0.0073–0.0847)

* *P<0.05*.

** *P<0.01*.

In the case of the DIY ABC approach, the simulations were based on the scenario of two populations that were diverged *t* generations ago from a shared ancestral population. The two populations seem to have diverged from 24.9 kyr BP (seven years generation time) to 17.8 kyr BP (five years generation time). In the popABC approach the time of divergence was estimated as 17,454 (8,485–26,523). Other parameters estimated by the popABC are available in Table S4. Both of these methods suggest that the genetic structure may have originated in the late Pleistocene, and that the two gene pools could have segregated before the last glacial maximum (LGM) in Icelandic waters.

## Discussion

### Sampling of siblings' effect

An accurate result from population genetic analyses requires a random, independent sample from each population of interest. However, within a population the genetic patterns can differ among demographic groups, and attention to this factor is important in order to attain an accurate conclusion regarding population genetic structure [79]. Parr are often sampled for population genetic research as they are more accessible and abundant than adult salmon, but parr samples may lead to false conclusions concerning population genetic structure due to the small numbers of successful breeders and biased breeding success [80]. If differences in allele frequencies between adult salmon and parr samples are solely due to skewed sampling of full-siblings, then removing the majority of full-siblings from the sample database should in turn remove these differences. The program STRUCTURE has been shown to be sensitive to samples with related individuals and can in such cases detect genetic structure within a sample where there is none [81]. Therefore it is important to take the sampling of sibling effect into account, and the stringency applied in this study was in accord with the suggestions of Anderson & Dunham [81]. For our dataset, the effect of removing siblings is most apparent in the case of Litla Laxa (12), where the average *F_ST_* compared to other samples is 0.027 lower. Removing siblings also caused the genetic distance between the two samples from the Blanda river system to drop from significant to non-significant.

### Genetic pattern, restricted gene flow, and discordance allozyme-microsatellite loci

Understanding the distribution and connectivity of populations has been one of the major challenges of recent decades, and has been recognised as a necessity for biodiversity conservation [82]. As such, indirect estimates of gene flow (i.e. the exchange of genes among populations due to the successful migrations of individuals) have received considerable attention in recent years. In marine organisms, populations that have been thought to be homogenous owing to a lack of obvious barriers have been shown to exhibit a clear genetic structure (i.e. restricted gene flow among populations on a small or large geographical scale [21], [24], [83], [84]).

The Atlantic salmon exhibits a complex biological cycle involving stages that inhabit both marine and freshwater environments. Several genetic studies have demonstrated its propensity to form distinct and reproductively isolated populations [37], [38], [85]–[87]. In the present study, substantial genetic differentiation was found between all collected samples except Olfusa (20) – Sog (16), and Svarta (18) – Blanda (1) (Table S2). Olfusa and Sog are indeed located in the Olfus river system - the river Sog is a tributary to Olfusa, with 13 km between sampling sites. Svarta is a tributary of Blanda, with only six kilometres between these sampling sites. The geographical proximity of these areas might have precluded any genetic differentiation among the samples collected in these rivers.

One noteworthy result from the present study was the high divergence between almost all samples collected, among all river systems, whereas a previous genetic study using allozyme loci and 22 sampling locations identical to the present research found that a total of 52 out of 231 possible pair-wise comparisons were non-significant [35]. This is in concordance with the assumption that microsatellite markers yield a higher resolution power than allozymes when identifying populations, due to the presence of many more alleles [88]. A study by King *et al.* [37] found that Icelandic salmon populations seem to be less diverse than other salmon populations originating from Europe, but more diverse than salmon populations in the USA and Canada (the average gene diversity for the Atlantic salmon populations in Iceland in this study was 0.69, compared to 0.60 in the USA and Canada and 0.74 for the rest of Europe [37]). Although there are only five out of twelve comparable microsatellite markers between the two studies, the same pattern was observed looking only at samples included in King *et al.* [37]. The highest pairwise *F_ST_* value between salmon populations in Iceland was 0.14 found between Kalfa (7) and Laxa Adaldal (9). Kalfa appeared to be a very unique sample and was highly differentiated from all other samples with a minimum *F_ST_* value of 0.075. If we excluded this sample, the *F_ST_* value range in this study was 0–0.116 and was very similar to what was observed within salmon populations in the UK [87]. The hierarchical genetic clustering method revealed a population structure to a relatively fine spatial scale in our population complex. This pattern concurred very well with the D_A_ distance phylogenetic tree and was geographically relevant, suggesting the effect of restricted gene flow was due to isolation by distance. The consequent predicament was a question of the extent to which to pursue the hierarchical analysis. In this case, we stopped at the second and third level, as we concluded that this sufficiently described the eight geographically major stock units of Iceland. Restricted gene flow can, of course, continue to act within some of the eight major genetic groups, but further subdivision (e.g. the within river structure of the Olfus river system) can be determined using the pairwise *F_ST_* comparison (Table S2). It should be noted that although we reduced the likelihood of false discovery by removing sibling groups from our samples, caution should be applied when interpreting *F_ST_* values, as they can be inflated by the effect of family structures between streams [89] or the possibility of low intra-stream heterozygosity [90].

In all, the present study corroborated previous genetic studies performed on Atlantic salmon by suggesting high levels of genetic differentiation between populations both within river systems [35], [38] and on a larger geographical scale [35], [37], [85]–[87]. Although historical climatological conditions in Iceland could explain the observed genetic pattern (see below), the present results suggest that gene flow tends to be restricted among Icelandic populations of Atlantic salmon.

### When climatology meets genetics

The glaciation and interglaciation periods of the Pleistocene have considerably shaped the distribution and connectivity of contemporary populations [10]–[12], [91]–[94]. Indeed, one of the major concerns about the genetic structure detected with microsatellite loci is that contemporary levels of differentiation might reflect a historical restriction of gene flow, rather than the current isolation of populations. Emerging evidence has shown historical signals embedded in microsatellite loci [4]–[6], [10], [11], [20], especially in Icelandic waters [22]–[24], [95] in which populations of marine organisms have colonized the ice-free environments following the LGM, examples of which are composed of relatively young populations on an evolutionary time scale.

**Figure 4:**
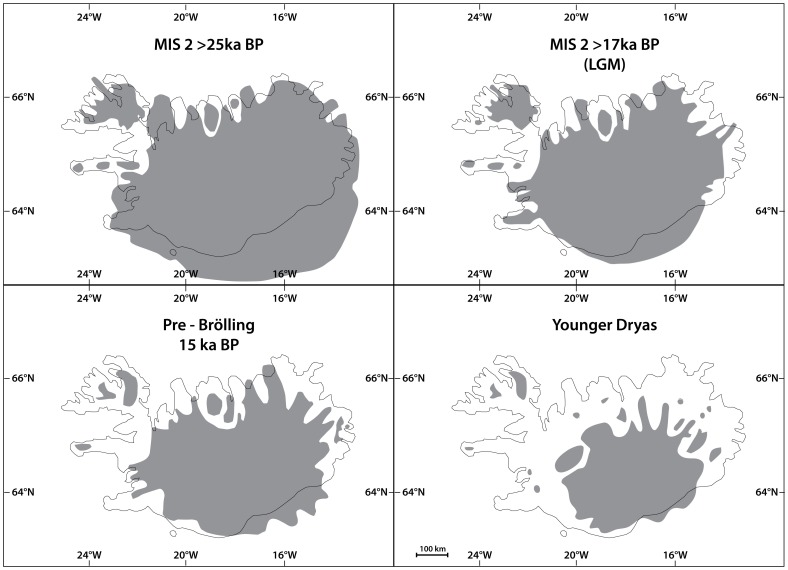
Deglaciation of Iceland redrawn from Van Vliet-Lanoë *et al.* [17]. The ice-sheet is represented by the shadowed area.

Although the complex biological cycle of the Atlantic salmon may be the primary source of potential genetic signals, the past geological and climatological history of Iceland may in part have been the origin of the contemporary genetic structure of salmon populations. In Iceland, most of the shelf water was covered by an ice-cap during the LGM; although scientists do not fully agree on the exact dates that the coastline of Iceland was deglaciated, they generally do agree upon the order of deglaciation in different areas. It has been suggested that this event started circa 17.5–15.4 cal. kyr BP and that by 15.4–14.6 cal. kyr BP the glaciers subsequently retreated well inside the coastline [27]. The order of events during the ice cap deglaciation of Iceland is demonstrated in Figure 4, on the time scale from 25 ka BP to the Younger Dryas. Based on these particular studies performed in Iceland, the first viable and ice-free environment for migrating salmon was likely to be located at Breiðafjörður in the northwest of Iceland (Figure 1). Thereafter, the ice-cap progressively retreated from the north and east of Iceland, before the final retreat occurred in the southwest and south of Iceland (Figure 4). Although Iceland is small compared to other areas where a latitudinal gradient in colonization would be expected, the icecap retreat model in Iceland is unusual as it follows a north to south pattern dissimilar to that of most other deglaciating areas [25], and the residues of this pattern are still visible when examining the modern-day glacial distribution within Iceland.

When considering neutral genetic markers the *F*-model assumes that a linkage equilibrium and Hardy-Weinberg equilibrium (HWE) exists within populations, such that genetic drift is the only force acting upon the population. With time, genetic drift will increase the population *F*-value, as was demonstrated by Group 2 having a higher *F*-value than Group 1. One possible interpretation of these results is that Group 2 was possibly colonized from Group 1 [60], or alternatively represents a second colonization event from another “source” population. However, other alternative processes might explain these results such as Holocene bottleneck and a smaller effective population size in the southern region (Group 2).

The genetic analyses, i.e. the existence of two main genetic clusters and the higher *F*-value in the south, do not provide any direct support for an initial colonisation in the north but are nevertheless consistent with the ice-cap retreat model in Iceland (and that the northern region was deglaciated first). When considering the deglaciation model of Iceland it is highly probable that pioneer salmon colonized the northern region before the southern region. The first available habitat for salmon in Iceland after the LGM is located in the Northwest region (Breiðafjörður) approximately 17.5 ka BP. From there suitable habitats were consecutively formed as the progression of deglaciation went north and east. The estimated time of divergence (DIYABC and popABC) demonstrated the existence and timing of subdivision between the northern and southern populations, and suggested that pioneer salmon were arriving in Iceland at a time when the only viable habitats were in the northern region (Breiðafjörður). Therefore, when we considered the available deglaciation model for Iceland, the presented genetic results seemed to be consistent with an initial settlement of Atlantic salmon in the northern region of Iceland.

The proposed order of settlement offers the possibility of three settlement scenarios: First, that the colonizing salmon inhabit the northern region and subsequently colonize the south. Second, that the colonizing salmon inhabit the north of Iceland and a second colonization event from the same population of origin occurs in the southern area. Third, that the colonizing salmon inhabit the north of Iceland and a second colonization event from a different refugium occurred in the southern area. Although we cannot distinguish between the suggested three settlement scenarios, our results suggest that the glaciation event in Iceland has imprinted the present day genetic structure of Atlantic salmon.

Although a three cluster scenario suggesting a colonization process consisting of multiple events cannot be completely ruled out, the current analysis better support the two cluster scenario. The additional within-river groups detected within each original group (Group 1 and Group 2) during our three levels of hierarchical structure analysis, and confirmed by the consensus neighbour joining tree of pairwise D_A_ [63] which is expected to produce the best branching tree pattern [96], might reflect more recent historical colonisation of river systems and current drift events due to recent isolation of populations (well after the colonisation). It might therefore represent the step-by-step colonisation of the different river systems, and more recent isolation events. Although Icelandic salmon populations are highly reproductively isolated, there is a possibility of gene flow between the two population groups - the DIYABC method does not take this into account, but this limitation is met by adding an analysis using the popABC program which allows for the modelling of gene flow between groups. Furthermore, minor inter-group gene flow might affect the estimates by underestimating the divergence time, but neither of these properties affect the glacial period in question.

### When history explains the exceptions

The rivers Ellidaa (25), Leirvogsa (11), and Langa (23) seemed to be misplaced in these ice-free environment re-colonisation events; however, a closer inspection of Icelandic volcanic history and of contemporary human activity offered explanations for these anomalies.

The lava field, Leitárhraun, was formed in a volcanic eruption 5,200 years ago [97], and stretches from the Bláfjöll down to Elliðavatn lake, where it formed the rootless volcanic cones, Rauðhólar, and from there it ran down to what has become the present river channel of Ellidaa (25) [98] (see insert on Figure 1). No pioneer salmon in Ellidaa (25) could have survived this natural event, therefore we suggest that a re-colonization event occurred, possibly shifting the river from its presumed geographical origin. Unknown human activity that might have caused this seems very unlikely but cannot be completely ruled out.

Regarding Leirvogsa (11), the river was probably inaccessible for salmon in its original state as there were cascades near the estuary. However, after the cascades were artificially made accessible to enhance rod fishery, the river became known for its sea-run brown trout. Today, Leirvogsa (11) is a river with a healthy salmon population since the river became stocked with parr and smolts originating from Ellidaa (25).

In Langa, (23) there are two waterfalls at the estuary - Sjávarfoss and Skuggafoss - that were possibly impassable for salmon. Skuggafoss waterfall proved especially difficult for salmon to pass until a fishway was built in the 1950s [99]. In the 1960's and 1970's the river was stocked with salmon parr and smolt, which originated from stocks of various origins. Four additional fishways were installed in waterfalls further upstream, thereby increasing the salmon habitat from 13 km to 26 km. This high level of human intervention could easily have altered the genetic signature of this river from its logical position in the gene pool of Icelandic salmon.

### Conclusion

The present study has yielded two important findings. Initially, that microsatellite loci revealed a significant genetic signal amongst the populations collected at 26 rivers distributed across the country, a result which suggested a restricted gene flow common to the sampled populations. Secondly, that the time of divergence between the two primary genetic groups detected was highly consistent with the ice-cap retreat model in Iceland, and that further analyses provide overwhelming evidence of a historical component to the observed genetic differentiation. The individual analysis of population structure strongly supports the presence of two genetically distinct groups, with a general trend of higher genetic variability in the northern areas in relation to that of the southern regions, thus substantiating the expectation of the existence of higher genetic variability in the potential initial area of colonization. The rivers Ellidaa (25), Leirvogsa (11) and Langa (23), however, do not seem to fit this re-colonisation route hypothesis, although the volcanic history of Iceland and the history of human activity for the previous 60 years could be attributed responsibility for the displacement of these rivers in the presented salmon histories. In addition, the additional variation observed within each predominant group (Group 1 and Group 2) was likely due to more recent events of colonisation or population isolation, subsequent to the recolonisation event after the LGM. We therefore conclude that the observed genetic pattern at microsatellite loci for the Atlantic salmon in Icelandic rivers serves as additional evidence of the relative immaturity of Icelandic fish populations, on account of the re-colonisation of this relatively young environment following the Last Glacial Maximum.

### Data archiving

Data available from the Dryad Digital Repository: http://doi.org/10.5061/dryad.60kk5.

## Supporting Information

Table S1 **Microsatellite loci characteristics assessed as repeat motif (RM), number of alleles (n), allelic richness (Ar), allelic size range (size range), gene diversity (Measured as expected heterozygosity, He) and overall** ***F*** **_ST_ and** ***R*** **_ST_.**
(DOCX)

Table S2 **Pairwise** ***F*** **_ST_ values (below diagonal) and their corresponding p-values for 0.01 significance level (above diagonal) for all sample pairs, as calculated in Arlequin.**
(DOCX)

Table S3 **Hierarchical analysis of variance (AMOVA) among samples of Atlantic salmon grouped into the two** ***a priori*** **groups detected with STRUCTURE.** The variance among groups relative to the total variance, the variance among samples within groups and the variance among samples relative to the total variance are presented. The source of variation from among groups, among samples within groups, and within samples is given as a percentage for each comparison. All F-values were highly significant (P<0.0001).
(DOCX)

Table S4 **The estimation from popABC with 95% confidence interval in parenthesis for splitting time (T), effective population sizes (N1, N2, NA) and migration rates (m1, m2).**
(DOCX)

Figure S1 **A PCA plot based on the** ***F*** **_ST_ values.** The two clusters of populations represent the two genetic clusters referred to throughout the text (Group 1 and Group 2).
(DOCX)

Figure S2 **A graphical output of the LOSITAN analysis for the two primary populations were heterozygosity (** ***He*** **) is on the x-axis and the** ***F_ST_*** **value is on the y-axis.** All loci fall within the simulated confidence area for neutral loci (grey area).
(DOCX)

Figure S3 **Clustering assignment of 26 salmon populations with STRUCTURE for K = (2, 3, and 4) with and without location information respectively.** Individuals are represented by a single vertical column divided into K colours. Each colour represents one cluster, and the length of the coloured segment corresponds to the individual's estimated proportion of membership in that cluster.
(DOCX)
