# Supplementary material for: Present-Day Genetic Structure of Atlantic Salmon (Salmo salar) in Icelandic Rivers and Ice-Cap Retreat Models
Source: PLoS One. 2014 Feb 3;9(2):e86809. doi: 10.1371/journal.pone.0086809 (PMC3911922; doi:10.1371/journal.pone.0086809)
Supplement: Table S1 — Microsatellite loci characteristics assessed as repeat motif (RM), number of alleles (n), allelic richness (Ar), allelic size range (size range), gene diversity (Measured as expected heterozygosity, He) and overall F ST and R ST. (DOCX) [file pone.0086809.s001.docx]

**Table S1.** Microsatellite loci characteristics assessed as repeat motif (RM), number of alleles (n), allelic richness (Ar), allelic size range (size range), gene diversity (Measured as expected heterozygosity, He) and overall *F*_ST_ and *R*_ST_.

| Locus | RM | n | Ar | Size range | He | *F*_ST_ | *R*_ST_ |
| --- | --- | --- | --- | --- | --- | --- | --- |
| Ssa14 | 2 | 2 | 2 | 147-151 | 0.2819 | 0.054 | 0.054 |
| Ssa171 | 4 | 13 | 9.99 | 207-259 | 0.7694 | 0.073 | 0.138 |
| SSa197 | 4 | 21 | 14.93 | 174-270 | 0.8459 | 0.052 | 0.046 |
| Ssa202 | 4 | 9 | 8.79 | 244-276 | 0.7711 | 0.045 | 0.042 |
| Ssa289 | 4 | 5 | 4.41 | 118-128 | 0.4851 | 0.075 | 0.047 |
| SSsp1605 | 4 | 10 | 6.72 | 223-263 | 0.6675 | 0.066 | 0.023 |
| SSsp2201 | 4 | 22 | 17.45 | 259-351 | 0.893 | 0.036 | 0.065 |
| SSsp2210 | 4 | 12 | 9.81 | 130-174 | 0.7467 | 0.081 | 0.094 |
| Sp2216 | 4 | 15 | 9.36 | 209-273 | 0.7405 | 0.057 | 0.020 |
| SSsp3016 | 4 | 13 | 8.99 | 91-139 | 0.6891 | 0.046 | 0.038 |
| SsaD144 | 4 | 32 | 24.41 | 122-246 | 0.8871 | 0.053 | 0.132 |
| Ssa157 | 4 | 25 | 17.68 | 316-412 | 0.8854 | 0.032 | 0.002 |
| SsaF43 | 2 | 10 | 4.65 | 108-130 | 0.2651 | 0.079 | 0.109 |
| SSspG7 | 4 | 16 | 8.81 | 128-204 | 0.7254 | 0.085 | 0.075 |
