# Supplementary material for: Present-Day Genetic Structure of Atlantic Salmon (Salmo salar) in Icelandic Rivers and Ice-Cap Retreat Models
Source: PLoS One. 2014 Feb 3;9(2):e86809. doi: 10.1371/journal.pone.0086809 (PMC3911922; doi:10.1371/journal.pone.0086809)
Supplement: Table S2 — Pairwise F ST values (below diagonal) and their corresponding p-values for 0.01 significance level (above diagonal) for all sample pairs, as calculated in Arlequin. (DOCX) [file pone.0086809.s002.docx]

**Table S2.** Pairwise *F*_ST_ values (below diagonal) and their corresponding p-values for 0.01 significance level (above diagonal) for all sample pairs, as calculated in Arlequin.

+ = p < 0.000001
