# Supplementary material for: Present-Day Genetic Structure of Atlantic Salmon (Salmo salar) in Icelandic Rivers and Ice-Cap Retreat Models
Source: PLoS One. 2014 Feb 3;9(2):e86809. doi: 10.1371/journal.pone.0086809 (PMC3911922; doi:10.1371/journal.pone.0086809)
Supplement: Table S3 — Hierarchical analysis of variance (AMOVA) among samples of Atlantic salmon grouped into the two a priori groups detected with STRUCTURE. The variance among groups relative to the total variance, the variance among samples within groups and the variance among samples relative to the total variance are presented. The source of variation from among groups, among samples within groups, and within samples is given as a percentage for each comparison. All F-values were highly significant (P<0.0001). (DOCX) [file pone.0086809.s003.docx]

**Table S3.** Hierarchical analysis of variance (AMOVA) among samples of Atlantic salmon grouped into the two *a priori* groups detected with STRUCTURE. The variance among groups relative to the total variance, the variance among samples within groups and the variance among samples relative to the total variance are presented. The source of variation from among groups, among samples within groups, and within samples is given as a percentage for each comparison. All F-values were highly significant (P<0.0001).

| Type of variation | df | Variance components | % Variation | Fixation index | *P*-value |
| --- | --- | --- | --- | --- | --- |
| Among groups | 1 | 0.14795 | 3.00 | 0.0300 | <0.000001 |
| Among samples within groups | 24 | 0.19966 | 4.04 | 0.0417 | <0.000001 |
| Within samples | 4030 | 4.58879 | 92.96 | 0.0704 | <0.000001 |
| Total | 4055 | 4.93639 | 100 |  |  |
