# Supplementary material for: Present-Day Genetic Structure of Atlantic Salmon (Salmo salar) in Icelandic Rivers and Ice-Cap Retreat Models
Source: PLoS One. 2014 Feb 3;9(2):e86809. doi: 10.1371/journal.pone.0086809 (PMC3911922; doi:10.1371/journal.pone.0086809)
Supplement: Table S4 — The estimation from popABC with 95% confidence interval in parenthesis for splitting time (T), effective population sizes (N1, N2, NA) and migration rates (m1, m2). (DOCX) [file pone.0086809.s004.docx]

**Table S4.** The estimation from popABC with 95% confidence interval in parenthesis for splitting time (T), effective population sizes (N1, N2, NA) and migration rates (m1, m2).

| **Parameter** | **Estimate with 95% confidence interval** |
| --- | --- |
| Splitting time (T): | 17,454 (8485 – 26,523) |
| Effective population size (N1): | 9775 (4749 – 15,219) |
| Effective population size (N2): | 12,903 (6230 – 19,560) |
| Effective population size (NA): | 12,889 (7410 – 17,035) |
| Migration rate (m1): | 0.001206 (0 - 0.002943) |
| Migration rate (m2): | 0.001729 (0.0001856 - 0.003258) |
