# Supplementary material for: Present-Day Genetic Structure of Atlantic Salmon (Salmo salar) in Icelandic Rivers and Ice-Cap Retreat Models
Source: PLoS One. 2014 Feb 3;9(2):e86809. doi: 10.1371/journal.pone.0086809 (PMC3911922; doi:10.1371/journal.pone.0086809)

**Figure S1.** A PCA plot based on the *F*_ST_ values. The two clusters of populations represent the two genetic clusters referred to throughout the text (Group 1 and Group 2).


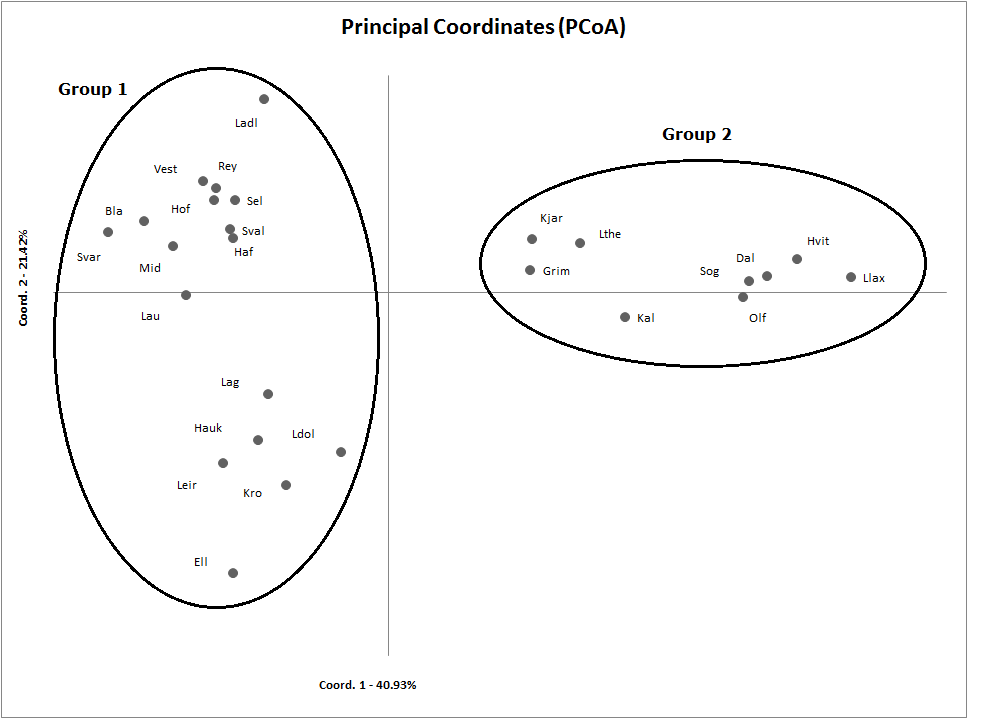

Supplement: Figure S1 — A PCA plot based on the F ST values. The two clusters of populations represent the two genetic clusters referred to throughout the text (Group 1 and Group 2). (DOCX) [file pone.0086809.s005.docx]
