# Supplementary material for: Present-Day Genetic Structure of Atlantic Salmon (Salmo salar) in Icelandic Rivers and Ice-Cap Retreat Models
Source: PLoS One. 2014 Feb 3;9(2):e86809. doi: 10.1371/journal.pone.0086809 (PMC3911922; doi:10.1371/journal.pone.0086809)

**Figure S2.** A graphical output of the LOSITAN analysis for the two primary populations were heterozygosity (*He*) is on the x-axis and the *F_ST_* value is on the y-axis. All loci fall within the simulated confidence area for neutral loci (grey area).


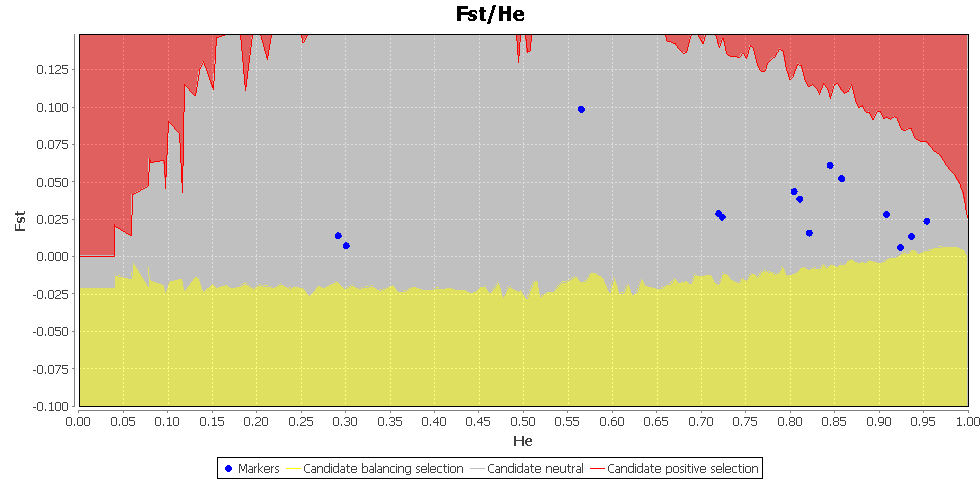

Supplement: Figure S2 — A graphical output of the LOSITAN analysis for the two primary populations were heterozygosity ( He ) is on the x-axis and the FST value is on the y-axis. All loci fall within the simulated confidence area for neutral loci (grey area). (DOCX) [file pone.0086809.s006.docx]
