# Supplementary material for: Present-Day Genetic Structure of Atlantic Salmon (Salmo salar) in Icelandic Rivers and Ice-Cap Retreat Models
Source: PLoS One. 2014 Feb 3;9(2):e86809. doi: 10.1371/journal.pone.0086809 (PMC3911922; doi:10.1371/journal.pone.0086809)

**Figure S3.** Clustering assignment of 26 salmon populations with STRUCTURE for K = (2, 3, and 4) with and without location information respectively. Individuals are represented by a single vertical column divided into K colours. Each colour represents one cluster, and the length of the coloured segment corresponds to the individual’s estimated proportion of membership in that cluster.


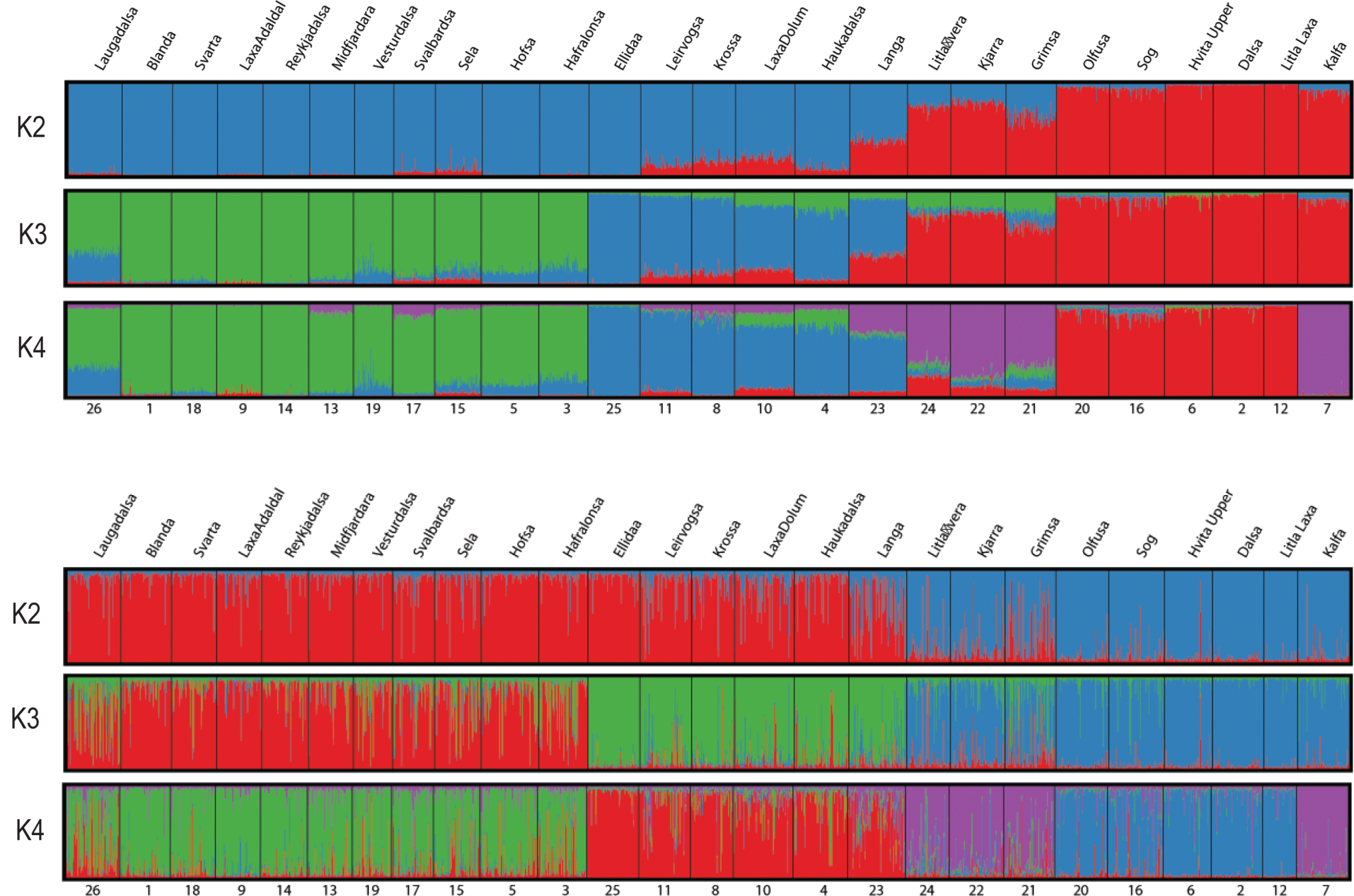

Supplement: Figure S3 — Clustering assignment of 26 salmon populations with STRUCTURE for K = (2, 3, and 4) with and without location information respectively. Individuals are represented by a single vertical column divided into K colours. Each colour represents one cluster, and the length of the coloured segment corresponds to the individual's estimated proportion of membership in that cluster. (DOCX) [file pone.0086809.s007.docx]
